# Chitosan Nanoparticles Enhance the Antiproliferative Effect of Lapachol in Urothelial Carcinoma Cell Lines

**DOI:** 10.3390/pharmaceutics17070868

**Published:** 2025-07-02

**Authors:** Tatiane Roquete Amparo, Kamila de Fátima da Anunciação, Tamires Cunha Almeida, Lucas Resende Dutra Sousa, Viviane Flores Xavier, Janaína Brandão Seibert, Ana Paula Moreira Barboza, Paula Melo de Abreu Vieira, Orlando David Henrique dos Santos, Glenda Nicioli da Silva, Geraldo Célio Brandão

**Affiliations:** 1 Postgraduate Program on Pharmaceutical Sciences, Federal University of Ouro Preto, Ouro Preto 35402-173, Brazil; lucasresendedutrasousa@gmail.com (L.R.D.S.); viviane.flores.xavier@gmail.com (V.F.X.); orlando@ufop.edu.br (O.D.H.d.S.); nicioli@ufop.edu.br (G.N.d.S.); celiobrandao@ufop.edu.br (G.C.B.); 2 Postgraduate Program on Biological Sciences, Federal University of Ouro Preto, Ouro Preto 35402-173, Brazil; kamila.fatima10@gmail.com (K.d.F.d.A.); paula@ufop.edu.br (P.M.d.A.V.); 3 Laboratory of Pain and Signaling, Butantan Institute, São Paulo 05503-900, Brazil; tamires-cunha@hotmail.com; 4 Natural Products Laboratory, Department of Chemistry, Federal University of São Carlos, Rod. Washington Luiz, São Carlos 13565-905, Brazil; jana_seibert@hotmail.com; 5 Department of Physiscs, Federal University of Ouro Preto, Ouro Preto 35402-173, Brazil; ana.barboza@ufop.edu.br

**Keywords:** lapachol, bladder cancer, nanoemulsion, nanospheres, chitosan

## Abstract

**Backgroud/Objectives:** Lapachol is a naturally occurring prenylated naphthoquinone with antiproliferative effects. However, its clinical application remains limited due to several factors, including poor water solubility, low bioavailability, and adverse effects. The development of chitosan-based nanoparticles holds promise in overcoming these challenges and has emerged as a potential nanocarrier for cancer therapy, including bladder cancer. The objective of this study was to develop and evaluate the effects of chitosan nanoparticles on bladder tumor cell lines. **Methods:** The nanoemulsion was prepared using the hot homogenization method, while the chitosan nanoparticles were obtained through the ionic gelation technique. The nanoformulations were characterized in terms of particle size and polydispersity index (PDI) using photon correlation spectroscopy, and zeta potential by electrophoretic mobility. Encapsulation efficiency was determined by ultracentrifugation, and the drug release was analyzed using the dialysis method. The antineoplastic potential was assessed using the MTT assay, and the safety profile was assessed through ex vivo analysis. Cellular uptake was determined by fluorescence microscopy. **Results:** The study demonstrated that both the chitosan-based nanoemulsion and nanospheres encapsulating lapachol exhibited appropriate particle sizes (around 160 nm), high encapsulation efficiency (>90%), and a controlled release profile (Korsmeyer–Peppas model). These nanoemulsion systems enhanced the antiproliferative activity of lapachol in bladder tumor cells, with the nanospheres showing superior cellular uptake. Histopathological analysis indicated the safety of the formulations when administered intravesically. **Conclusions:** The results suggest that chitosan nanoparticles may represent a promising alternative for bladder cancer treatment.

## 1. Introduction

2-Hydroxy-3-(3-methylbut-2-en-1-yl)-naphthalene-1,4-dione, known as lapachol, is a naturally occurring para-benzoquinone-containing hydroxy compound [1]. The first identified natural source of lapachol was *Tabebuia impetiginosa*, a member of the Bignoniaceae family, commonly known as “red lapacho.” *T. impetiginosa* is native to the Amazon rainforest and other parts of Latin America [2], and is widely used in traditional South American phytomedicine [3]. In addition to *T. impediginosa*, lapachol has been identified in other species of the Bignoniaceae family, including *Handroanthus serratifolius* [4], which is endemic to Brazil and commonly referred to as “ipê-amarelo” [5]. Lapachol can be extracted in satisfactory yields through an acid-base extraction method followed by recrystallization [6].

Numerous studies have previously documented the pharmacological activities of lapachol, including anticancer, antimalarial, analgesic, antiviral, antiparasitic, antipsoriatic, antioxidant, antimicrobial, anti-inflammatory, and pesticidal effects [7]. In cancer research, lapachol has received considerable attention for its well-documented antiproliferative effects, demonstrated in various in vitro and in vivo tumor models [8]. It has shown efficacy against multiple cancer types, including esophageal [9], lung [10], breast [11], fibrosarcoma [12], and glioma [13]. Its antineoplastic mechanism involves caspases and PARP activation, cytochrome c and BAX induction [9], NF-κB pathway activation [10], matrix metalloproteinases inhibition [12], and targeting of DNA topoisomerases I and II [13].

Despite the promising anticancer potential of lapachol, its clinical application is hindered by factors such as poor water solubility, low bioavailability, and adverse effects [11,14]. These limitations may be addressed through nanotechnology-based drug delivery systems. Chitosan-based nanoparticles have emerged as a promising nanocarrier for cancer treatment, including bladder cancer [15].

Chitosan can be employed in various nanocarrier formats, with nanospheres and nanoemulsions standing out for their utility [16]. Nanoemulsions are heterogeneous oil-in-water or water-in-oil dispersions stabilized by emulsifying agents and are frequently used to deliver poorly water-soluble anticancer drugs [16]. For instance, nanoemulsions containing cisplatin [17] and resveratrol [18] have been shown to improve the anti-bladder cancer efficiency of these agents.

Nanospheres are a type of polymeric nanoparticle composed of a continuous polymeric matrix capable of entrapping or adsorbing drugs on its surface [19]. Chitosan nanospheres have been used to deliver chemotherapeutics such as paclitaxel, mitomycin C, and cisplatin for bladder cancer treatment [20,21,22], showing effective antitumor activity and reduced toxicity [23].

Nanostructured formulations containing lapachol, including nanoemulsions and liposomes, have already been developed, demonstrating enhanced antitumor activity [11,14]. A study reported that lapachol-loaded nanoemulsions enhanced antitumor activity both in vitro and in vivo, with no signs of toxicity in bladder cancer models [11]. Additionally, lapachol-loaded liposomes have improved anti-glioma efficacy by prolonging systemic circulation and increasing brain uptake [14].

However, the development of chitosan-based lapachol nanoparticles and their impact on bladder cancer treatment has not yet been documented. Bladder cancer was selected as the target in this study due to its high prevalence among urinary tract malignancies, its complex biological behavior, and its high recurrence rate, all of which present substantial challenges to clinical management [24]. Furthermore, no studies have been identified in the literature evaluating lapachol-loaded chitosan nanoparticles in bladder cancer cell lines.

Chitosan-based formulations have shown potential for treating both non-muscle-invasive bladder cancer (via intravesical instillation) and muscle-invasive bladder cancer (via systemic administration) [25]. In intravesical applications, chitosan nanoparticles can penetrate deeper bladder wall layers and enable sustained drug release, reducing the need for repeated administrations [25,26,27]. Oral administration of chitosan nanoparticles has also been shown to enhance drug bioavailability and reduce systemic side effects, demonstrating both efficacy and safety [25,28,29,30].

Given the relevance of chitosan in bladder cancer treatment and its applicability in both nanoemulsions and nanospheres, these systems were selected for lapachol encapsulation. Accordingly, the objective of this study was to develop and evaluate the effects of chitosan nanoparticles loaded with lapachol on bladder tumor cell lines.

## 2. Materials and Methods

### 2.1. Materials

Deacetylated shrimp shell-derived chitosan (minimum 75% deacetylation), dimethyl sulphoxide (DMSO), glycerol, sodium triphosphate (TPP), Dulbecco’s Modified Eagle Medium (DMEM), fluorescein isothiocyanate (FITC), 4′,6-diamidino-2-phenylindole dihydrochloride (DAPI), and fluoromount solution were purchased from Sigma Aldrich (St. Louis, MO, USA). Polysorbate 80 (Tween 80) and acetic acid were purchased from Synth (São Paulo, Brazil). Lapachol was extracted from *Handroanthus serratifolius* trunkwood by Brandão et al., 2018 [4].

### 2.2. Preparation and Characterization of the Nanostructured Formulations

The formulations were prepared using chitosan derived from deacetylated shrimp shells dissolved in ultrapurified water containing 0.5% acetic acid, resulting in a concentration of 0.1%. Lapachol was dissolved in DMSO. The nanoemulsion was prepared using the hot homogenization method, while chitosan nanoparticles were obtained via the ionic gelation technique [11,31].

To obtain the nanoemulsion, the chitosan solution and the oil phase (soybean oil, surfactants, and lapachol dissolved in DMSO) were heated to 80 °C. The aqueous phase was then gradually added to the oil phase under continuous agitation using an Ultra-Turrax T-25 homogenizer (Ika Labortechnik, Staufen, Germany) at 20,000 rpm, and homogenization was maintained until the mixture cooled to 25 °C [11]. The final composition of the nanoemulsion was as follows: lapachol (0.1% *w*/*v*), glycerol (2.0% *w*/*v*), Polysorbate 80 (2.0% *w*/*v*), soybean oil (4.0% *w*/*v*), DMSO (2.0% *w*/*v*), and chitosan (0.1% *w*/*v*).

To obtain the nanospheres, 8 mL of the chitosan solution at 60 °C was added to a mixture of lapachol solubilized in DMSO and Polysorbate 80. This mixture was stirred using an Ultra-Turrax T-25 homogenizer (Ika Labortechnik, Germany) at 20,000 rpm. Subsequently, a 0.1% TPP solution was slowly added, and the stirring was maintained at 25,000 rpm for 20 min [31]. The final composition of the nanospheres was as follows: lapachol (0.1% *w*/*v*), Polyssorbate 80 (5.0% *w*/*v*), DMSO (2.0% *w*/*v*), and TPP (0.02% *w*/*v*).

#### 2.2.1. Dynamic Light Scattering (DLS) and Zeta Potential

The nanoformulations were characterized in terms of particle size and polydispersity index (PDI) using dynamic light scattering (DLS), and zeta potential using electrophoretic mobility, both measured with a Zetasizer (Malvern, model Zetasizer Nano series—Nano ZS, Malvern, UK. For size and PDI analysis, samples were diluted in ultrapure water (1:1000) and transferred to quartz cuvettes. For zeta potential measurement, the diluted samples were placed in a capillary cell. All measurements were performed in triplicate at a controlled temperature of 25 ± 2 °C.

#### 2.2.2. Atomic Force Microscopy (AFM)

Atomic force microscopy (AFM) characterization of the nanoparticles was performed using a Park XE7 scanning probe microscope (SPM) (Park Systems Corp., Swon, Republic of Korea), operating in intermittent contact mode. Silicon cantilevers (NSC35/ALBS) (MikroMash, Wetzlar, Germany) with spring constants of 5–15 Nm^−1^ and a tip radius of curvature of approximately 10 nm were used for sample imaging. Measurements were conducted under ambient humidity and temperature conditions. Samples were prepared using the spread-coating method, in which approximately 10 μL of each formulation was deposited onto a freshly cleaved mica substrate (1.0 × 1.0 cm) and dried with a nitrogen gas jet after 30 s. All AFM images were processed (leveling, profiling, and 3D rendering) using the open-source software Gwyddion 2.58 [32]. Geometrical diameters were measured from the topography images

#### 2.2.3. Fourier Transform Infrared (FTIR) Spectroscopy and High-Performance Liquid Chromatography with Diode Array Detection (HPLC-DAD)

Fourier Transform Infrared (FTIR) spectroscopy was used to analyze the nanoemulsions and nanospheres, both with and without lapachol, as well as isolated lapachol. Analyses were performed using a Shimadzu IRPrestige-21 FTIR spectrometer, with a background spectrum acquired as a negative control. Spectra were recorded in the range of 400 to 4000 cm^−1^, with a resolution of 4 cm^−1^ and 32 scans per sample [33].

High-performance liquid chromatography with diode array detection (HPLC-DAD) was conducted using a Waters Alliance system (Waters, Milford, MA, USA) equipped with a C18 column (Luna, 4.6 × 250 mm, 5 μm particle size; Phenomenex, Torrance, CA, USA), maintained at 30 °C. The mobile phase consisted of 20% (*v*/*v*) 0.1% formic acid in ultrapure water and 80% (*v*/*v*) methanol, under isocratic conditions. The flow rate was 1.0 mL/min, and the injection volume was 20 μL [11].

#### 2.2.4. Encapsulation Efficiency and Drug Release

The encapsulation efficiency (EE) was determined using the ultracentrifugation/ultrafiltration method in microtubes. A volume of 500 µL from each formulation was transferred to microtubes and subjected to centrifugation at 20,000× *g* for 30 min. The resulting eluates were collected, and the amount of free lapachol was quantified by spectrophotometry. EE was then calculated using the following equation:

$$EE=\frac{([total\;lapachol])-\left[free\;lapachol\right]}{\left[total\;lapachol\right]}\times 100$$

The dialysis method was employed to analyze the in vitro drug release [11]. The formulations were placed inside dialysis membrane (SnakeSkinTM Dialysis Tubing), which were then immersed in the receptor medium (ethanol 50% *v*/*v* in PBS, pH 6.2) under continuous stirring at 37 °C. Aliquots were withdrawn from the receptor medium at predetermined intervals (1, 2, 4, 6, 8, 24, and 48 h), and an equal volume of fresh medium was replaced to maintain sink conditions. To evaluate the drug-release kinetics, the following plots were generated: (1) cumulative percentage of drug release versus time to assess zero-order kinetics, (2) log cumulative drug remaining versus time for first-order kinetics, (3) cumulative percentage of drug release versus the square root of time to analyze diffusion-controlled release based on the Higuchi model; and (4) log cumulative percentage of drug release versus log time according to the Korsmeyer–Peppas model. The coefficients of determination (R^2^) for each model were compared to analyze the drug release kinetics. The formulation type, the R^2^ value closest to 1, and the angular coefficient value (*n*) (in the case of the Korsmeyer–Peppas model) were used as parameters to determine the best kinetic profile [34].

The lapachol concentration for encapsulation efficiency and in vitro drug release assays was quantified by spectrophotometry. Aliquots of 200 μL of lapachol solutions, ranging from 200 to 1000 μg/mL, were added in 96-well plates, and absorbance was measured at 482 nm using a Spectrophotometer—TECAN Nanoquant Infinite 200 Pro (Tecan Austria GmbH, Grodig, Austria). The analytical method was semi-validated to demonstrate the linearity, precision (repeatability), and accuracy [35].

### 2.3. Cytotoxicity Assay

The cytotoxicity of the formulations was evaluated using the MTT (3-[4,5dimethylthiazol-2-yl]-2,5-diphenyltetrazolium bromide) reduction assay [36]. Human urothelial carcinoma cells (RT4, J82, and T24), obtained from the Cell Bank of Rio de Janeiro, Brazil were cultured in DMEM supplemented with 10% fetal bovine serum, 100 U/mL penicillin G, 100 U/mL streptomycin, and 2.5 μg/mL amphotericin B, at 37° C in a 5% CO_2_ atmosphere. Cells were seeded at a density of 2 × 10^4^ cells per well in 96-well plates 24 h prior to the treatment. Subsequently, cells were exposed to the formulations (3.1 to 100 mg/mL) or free lapachol (3.1 to 100.0 µg/mL) for 48 h. The cytotoxic concentration causing 50% reduction in cell viability (CC_50_) was calculated relative to the untreated cells (control).

### 2.4. Cell Uptake

To evaluate cellular uptake, the formulations were prepared with fluorescein isothiocyanate (FITC) at a concentration of 2 mg/mL as a fluorescent marker. Cells (2 × 10^5^ cells/well) were seeded in chamber slides and treated with the formulations (12.5 µg/mL). After 4 h, the cells were washed with Hank’s solution and fixed with 4.0% paraformaldehyde. The cells were then washed and stained with DAPI (0.2 µg/mL), followed by a final wash. Slides were mounted using Fluoromount solution. Images were captured using a fluorescence microscope equipped with a 20×objective. Fluorescence quantification was performed using ImageJ software 1.50 (NIH, Bethesda, MD, USA) [37].

### 2.5. Ex Vivo Toxicity

Freshly extracted porcine urinary bladders were obtained from a slaughterhouse in a cold box and stored at 0 °C prior to retention studies. Defrosted tissues were carefully cut into approximately 1 × 1 cm^2^ sections without touching the internal mucosa, which were then used for experiments. With their mucosal surface facing upward, bladder tissue sections were mounted on glass slides and washed with PBS buffer. Aliquots of 10 µL of each formulation or deionized water (control) were applied onto the bladder mucosa (1 × 1 cm^2^). Samples were incubated for 4 h at 37 °C in a humidity chamber. After incubation, the bladder mucosa was washed to remove excess formulations and fixed in 4% neutral-buffered formalin. Tissue sections of 5 μm thickness were prepared following standard histological procedures, stained with hematoxylin–eosin (HE), and examined histopathologically using a light microscope. For each formulation, three different bladder tissue samples were analyzed to assess potential tissue damage in healthy mucosa [37].

### 2.6. Statistical Analysis

Normality of the data was assessed using the Shapiro–Wilk test. Results are presented as mean ± standard error and were analyzed by one-way analysis of variance (ANOVA) followed by Tukey’s or Dunnett’s post hoc test for multiple comparisons, using GraphPad Prism 8.0.1 software. A *p*-value of less than 0.05 was considered statistically significant.

## 3. Results and Discussion

### 3.1. Physicochemical and Morphological Characterization of the Nanoemulsion and Nanospheres

To assess the most effective system for anticancer activity, particularly against bladder cancer cells, lapachol was encapsulated in two distinct nanostructures: nanoemulsion and nanospheres. The selection of these nanocarriers was based on their common use with chitosan. Chitosan was chosen due to its status as a natural polymer that is biocompatible, biodegradable, and exhibits low toxicity [15]. Moreover, chitosan offers significant potential advantages in bladder cancer treatment because of its mucoadhesive properties [25].

The presence of chitosan supports the positive potential of the developed nanoformulations: the nanoemulsion and nanospheres exhibited zeta potentials of +22.8 ± 0.8 mV and +21.8 ± 1.6 mV, respectively. The zeta potential values for both formulations fall within the range reported in the literature and are considered stable (>20 mV) [38]. For instance, a study on chitosan nanoparticles loaded with hesperidin, a natural antitumor compound, reported a zeta potential of +21 mV [39]. The surface charge of nanoparticles is a crucial characteristic influenced by three main factors: the type of material used, the TPP–chitosan ratio, and the pH of the reaction [40]. Therefore, the similar zeta potentials observed for the nanoemulsions and nanospheres may be attributed to the use of the same material and reaction pH.

However, despite the presence of chitosan in both nanostructures, there are structural and compositional differences between the two systems. Lipid nanoemulsions are colloidal dispersions in which the particles consist of an oily core surrounded by surfactants [41]. In addition to these components, absorbent polymers, bioenhancers, and other excipients have been shown to enhance the pharmacological activities of nanoemulsions [41]. In contrast, nanospheres lack an oily core. Nanoparticles produced by ionic gelation are composed of chitosan chains crosslinked by an anionic crosslinker (in this case, TPP) [42]. In summary, the main distinction between the two formulations lies in the composition of their cores. Specifically, the nanoemulsion contains a distinct oil core, whereas the nanospheres consist of a continuous polymeric network.

**Figure 1:**
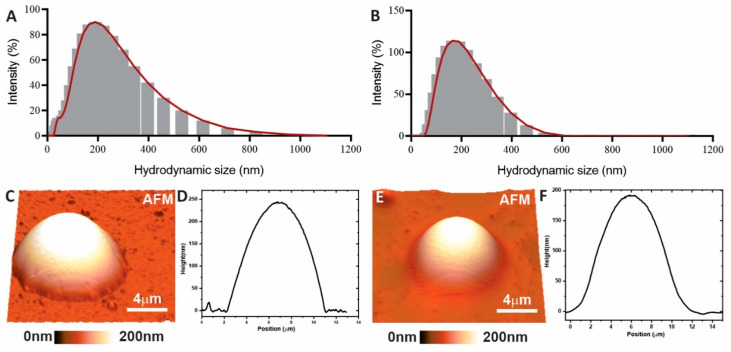
Particle size distribution histograms obtained from the dynamic light scattering (DLS) (**A**,**B**), images of atomic force microscopy (AFM) (**C**,**E**) with their respective profiles (**D**,**F**).

According to the DLS analyses, the developed nanoemulsion exhibited a particle size of 169.4 ± 1.5 nm and a PDI of 0.40 ± 0.10. The nanospheres showed similar dimensions, with an average size of 162.9 ± 14.4 nm and a PDI of 0.30 ± 0.06 (Figure 1A,B). Therefore, both formulations can be classified as nanometric with a homogeneous size distribution, meeting the desired parameters for a nanoparticle (size 100–400 nm and PDI < 0.5) [43].

The particle size was confirmed in AFM analyses (Figure 1C,D). Both formulations (nanospheres and nanoemulsion) exhibited rounded particles with an approximate size of 200 nm. The particle size of the lapachol-loaded formulations is favorable for bladder cancer treatment, as particles sized between 50 and 500 nm can enhance drug permeability through the urothelium. This is supported by Chen et al. 2020 [44], who reported that nanoemulsions averaging 200 nm improved the efficacy of gemcitabine and doxorubicin in bladder cancer treatment.

In comparison with other studies involving lapachol nanoformulations, Mendes Miranda et al. 2021 [11] developed a similar lapachol-loaded nanoemulsion with an average size of 174 ± 2 nm and a zeta potential of −17.9 ± 4.0 mV. On the other hand, the nanoemulsion containing lapachol developed by Rodrigues et al. (2018) exhibited a smaller size of 73.1 ± 2.1 nm and a zeta potential of −27.1 ± 2.2 mV [45]. Unlike these two studies, the formulation developed in the present work contains chitosan. The incorporation of chitosan altered the zeta potential due to the positive charge of the polymer, while the particle size remained similar to that reported by Mendes Miranda et al. 2021 [11]. The difference in particle size compared to Rodrigues et al. (2018) [45] can be attributed to the use of different oils and/or surfactants. To date, no data are available in the literature regarding lapachol-loaded chitosan nanospheres obtained by ionic gelation.

### 3.2. Encapsulation Efficiency and Drug Release

**Figure 2:**
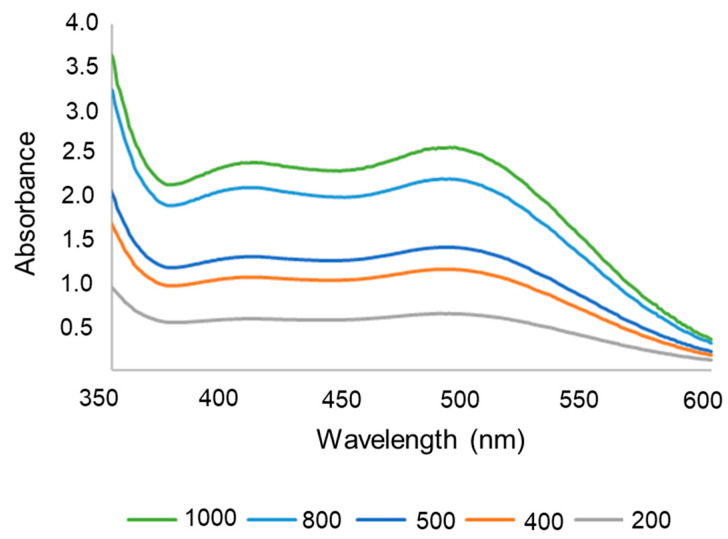
Ultraviolet spectrum of lapachol in PBS with 50% ethanol, pH 6.2, at 1000 to 200 µg/mL.

To evaluate the encapsulation efficiency and in vitro drug release of lapachol, its concentration was determined by spectrophotometry. The lapachol spectrum showed absorption maxima at 482 nm (Figure 2), consistent with the data reported by Segoloni and Di Maria (2018) at pH 6.2 [46]. Therefore, the wavelength of 482 nm was selected for lapachol quantification.

The quantification method was considered precise and accurate, demonstrated by low relative standard deviation (RSD) values of 5%, as shown in Table 1. Additionally, the method exhibited good linearity in the range of 100 to 1000 μg/mL, along with appropriate detection and quantification limits (Table 1). Consequently, this method was employed for quantifying lapachol in encapsulation efficiency and release assays.

The nanoemulsion and nanospheres exhibited encapsulation efficiencies of 94.95 ± 0.15% and 95.13 ± 0.21%, respectively. These results indicate that both chitosan-based nanoparticles have high encapsulation efficiencies (>90%) [47]. Similar findings have been reported in other studies involving the encapsulation of natural compounds in chitosan nanoparticles. For instance, nanoparticles containing ellagic acid for oral cancer treatment demonstrated an encapsulation efficiency of 94% [48].

**Figure 3:**
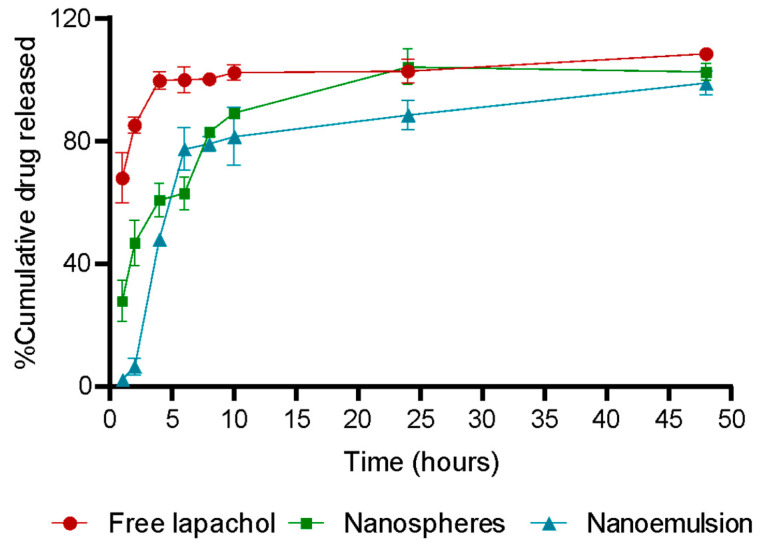
In vitro release of free lapachol, nanospheres, and nanoemulsion expressed as percentage of cumulative drug released.

Regarding the in vitro release, the results are presented as cumulative percentages of lapachol released over time, as shown in Figure 3. The release study was conducted at pH 6.2, which corresponds to the typical urine pH. Intravesical administration is considered a favorable treatment route for bladder cancer using chitosan nanoparticles. These nanoparticles enhance drug retention in the bladder and improve urothelial permeability due to their mucoadhesive properties [25]. Direct instillation of therapeutic agents into the bladder via catheter has been shown to increase treatment efficacy while minimizing systemic off-target side effects [49,50]. This is primarily because intravesical administration allows for higher drug concentrations at the tumor site [49,50].

The release of free lapachol occurred rapidly, with over 67% release within the first hour and nearly 100% after 4 h, characterizing an immediate release. In contrast, this rapid release was not observed in the nanospheres or the nanoemulsion. Approximately 28% of the lapachol encapsulated in the nanospheres was released during the first hour, indicating greater release control compared to free lapachol. After 6 h, around 63% was released, increasing to approximately 89% after 10 h, demonstrating a slower and sustained release profile. Lapachol release from the nanoemulsion was even slower. Only about 2% of the loaded drug was released in the first hour, and approximately 6% after two hours, suggesting that the nanoemulsion strongly restricts rapid initial release. However, after 4 h, the release rate increased, reaching nearly 45%, and the sustained release continued up to 10 h. Complete release of lapachol was observed only after 24 h.

It is essential to define the release kinetic model, as it helps to understand the mechanism by which the active ingredient is released from the formulation. This understanding directly influences the therapeutic efficacy of the formulation [51]. Table 2 presents the adjusted kinetic coefficient (R^2^) data.

In nanospheres, the Korsmeyer–Peppas model showed the best fit (R^2^ = 0.8820) with an n value of 2.621, characterizing a Super Case-II release mechanism. This suggests that lapachol release occurs primarily through polymer matrix relaxation and controlled erosion, alongside diffusion. Therefore, the release is governed by two distinct mechanisms: the manipulation of the polymer matrix and the movement of lapachol within the nanostructure [51]. For the nanoemulsion, the Korsmeyer–Peppas model showed a moderate fit (R^2^ = 0.7200) with an n value of 0.7354, indicating an anomalous transport mechanism, where drug release results from a combination of diffusion and structural reorganization of the system. Processes such as droplet fusion, drug redistribution between phases, and interactions with the external environment can influence the release profile [51]. The Korsmeyer–Peppas model has also been associated with the kinetics of other chitosan nanoparticles, such as those loaded with salicylic acid developed by Hassanpour and colleagues for breast cancer targeting [52].

### 3.3. Chemical Stability of Lapachol in Formulation Obtention Process

**Figure 4:**
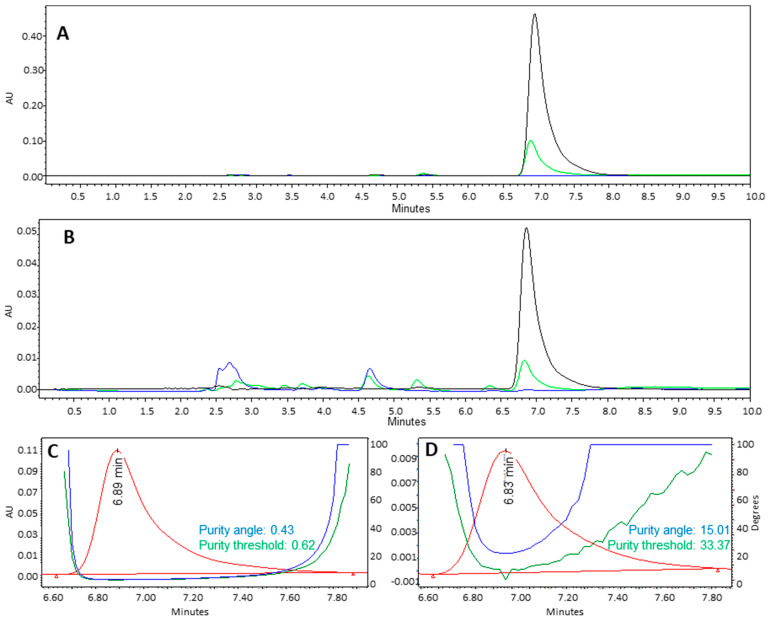
Chromatograms (extracted at 250 nm) of nanospheres (**A**) and nanoemulsion (**B**) in which standard lapachol is represented in black, lapachol-loaded nanoparticles in green, and empty nanoparticles in blue. Purity analysis of the lapachol peak (6.8 min) in nanospheres (**C**) and nanoemulsions (**D**).

To evaluate the stability of lapachol during the nanoparticle production process, high-performance liquid chromatography with diode array detection (HPLC-DAD) and infrared spectroscopy analyses were performed. The HPLC-DAD results showed no additional peaks in the lapachol-loaded formulations beyond those corresponding to the lapachol standard or the excipients present in the empty formulation (Figure 4A,B). Furthermore, the lapachol peak at 6.8 min demonstrated satisfactory purity, with a purity angle below the threshold value (Figure 4C,D).

**Figure 5:**
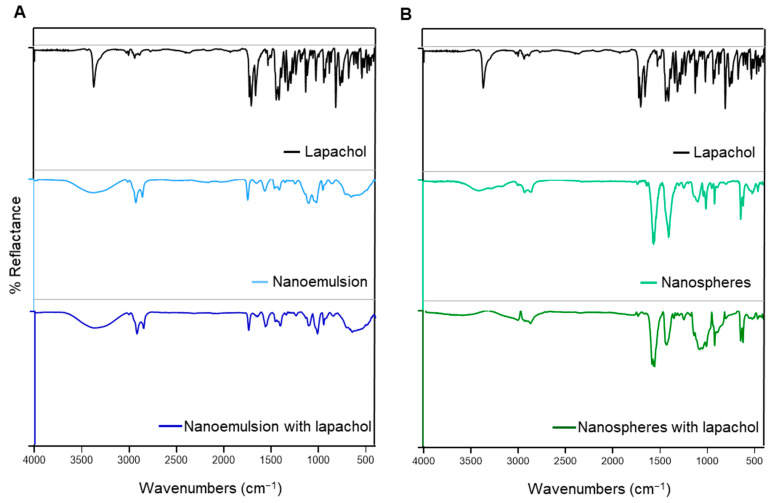
FTIR spectra for lapachol, chitosan nanoemulsion (**A**), and nanospheres (**B**) with and without lapachol.

The FTIR spectrum of lapachol showed bands characteristic of this compound: a narrow and strong band at 3348 cm^−1^ and 1660 cm^−1^, which can be attributed to the stretching of O–H groups and C1=O1, respectively; the bands at 2993, 2970, 2902, and 2850 cm^−1^ related to stretching of CH (C-12), CH 3 (14 and 15), and CH 2 (C-11) groups, respectively; and the band related to the CH scissor mode (1367 cm^−1^) and the out-of-plane vibrations of the aromatic rings (721 cm^−1^) (Figure 5) [53].

Spectra of the nanoemulsion and nanospheres revealed bands associated with the presence of chitosan: a 3400 cm^−1^ band attributed to stretching vibrations of -NH_2_ and -OH groups; 1650 and 1560 cm^−1^ bands ascribed to amide I and amide II, respectively; and 1100 and 1020 cm^−1^ bands due to skeletal vibrations involving O–C bonds (Figure 5) [54]. Due to the presence of oil and surfactants in NE and TPP in NS, differences between NE and NS were expected [33,54].

In formulations containing lapachol, no additional bands were observed compared to spectra of formulations without the active ingredient or the lapachol spectrum. Therefore, no bands related to the formation of other derivatives or interaction between lapachol and other constituents were observed. Considering the HPLC-DAD and FTIR results, it can be concluded that lapachol was not degraded during the nanoparticle production process.

### 3.4. Antiproliferative Activity

**Figure 6:**
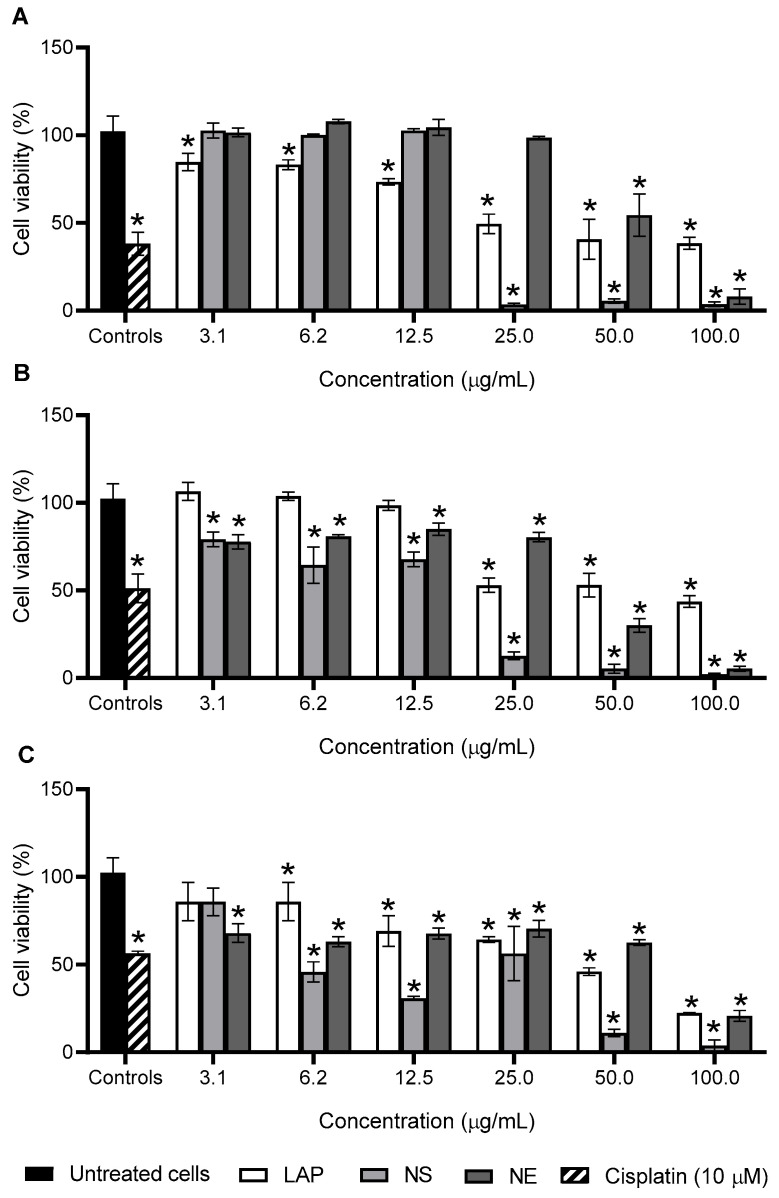
Cell viability of RT4 (**A**), J82 (**B**), and T24 (**C**) cell lines after 24 h, 48 h, and 72 h of treatment with free lapachol (LAP), nanoemulsion (NE), or nanospheres (NS). * *p* < 0.05 in relation to untreated cells by One-way ANOVA test followed by a Dunnett’s post-test.

The antiproliferative activity of the nanoemulsion and nanospheres was evaluated using three different cell lines (RT4, J82, and T24). The RT4 cell line originates from a low-grade bladder tumor, whereas J82 and T24 are derived from high-grade tumors. Low-grade bladder cancers are typically non-invasive tumors and present as superficial papillary growths, while high-grade tumors tend to be invasive and metastatic [55]. The treatments exhibited a concentration-dependent cytotoxic effect, as shown in Figure 6.

Notably, the highest concentrations of the formulations exhibited cytotoxic effects that were comparable to, or even surpassed, those of cisplatin (Figure 6). Despite the occurrence of resistance in some cases, cisplatin remains a cornerstone of chemotherapy regimens for patients with bladder cancer [56]. It is widely recognized as a potent antiproliferative agent used in the treatment of various malignancies [56].

A comparison of the treatments revealed that both nanoparticles significantly enhanced the cytotoxic activity of lapachol, as indicated by lower CC_50_ values compared with free lapachol across all tested cell lines (Table 3). In contrast, the nanoemulsion developed by Mendes Miranda et al. 2021 showed no significant difference in lapachol cytotoxicity against breast cancer cells (MDA-MB-231 and 4T1 cell lines) [11]. However, other studies have reported that chitosan nanoparticles can enhance the antiproliferative activity of various natural compounds, such as curcumin, in breast cancer cells [57]. When comparing the two formulations, the nanospheres exhibited significantly greater cytotoxicity, as evidenced by lower CC_50_ values (*p* < 0.05), suggesting superior antiproliferative activity (Table 3).

**Figure 7:**
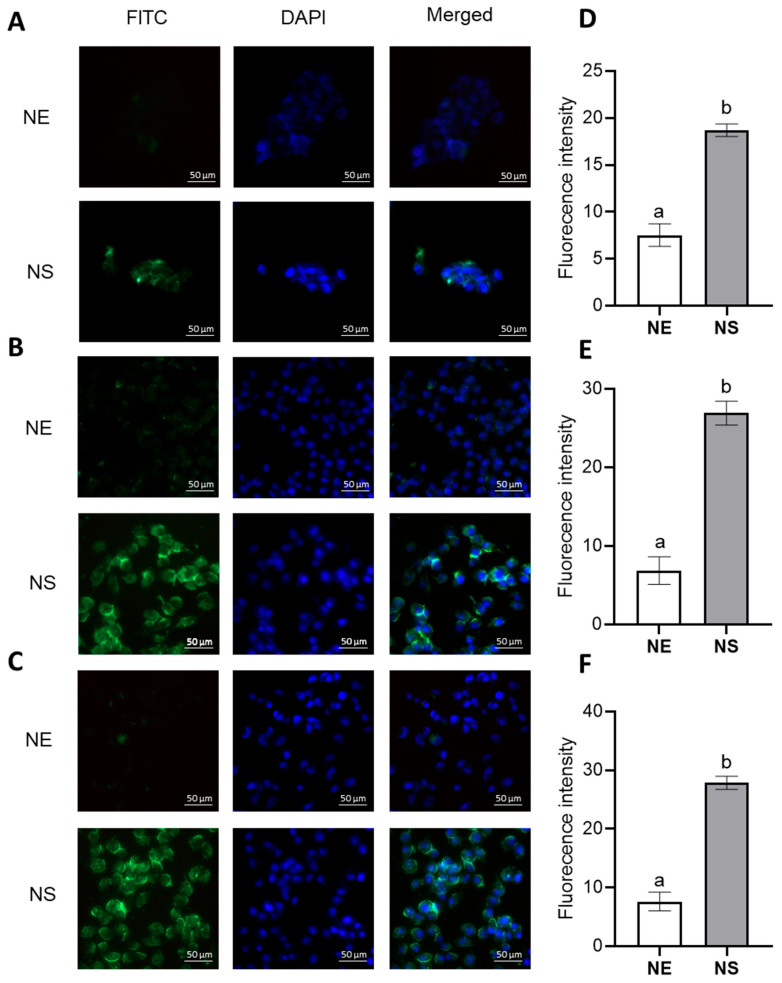
Cell uptake of the nanoemulsion (NE) and nanospheres (NS) by fluorescence microscopy for RT4 (**A**,**D**), J82 (**B**,**E**), and T24 (**C**,**F**) cell lines. In (**D**–**F**), the same lowercase letters indicate no significant statistical difference (*p* < 0.05) by t test.

The enhanced cellular toxicity observed with the nanospheres, compared to the nanoemulsion, may be attributed to their superior cell uptake, as demonstrated in Figure 7. The positive surface charge of the chitosan, the polymeric material used in both formulations, facilitates electrostatic interactions with the negatively charged cell membrane, thereby promoting internalization [58,59]. For example, Bhirud et al. (2025) demonstrated that chitosan nanoparticles enhanced the internalization of imatinib by approximately 20% in colon cancer cells [60]. Chitosan nanoparticles are typically internalized through active endocytic pathways, such as phagocytosis and pinocytosis, further supporting their effectiveness in drug delivery applications [59].

### 3.5. In Vitro Selectivity and Ex Vivo Toxicity

In addition to evaluating the antitumor efficacy, the safety of the formulations was assessed both in vitro using normal cells and ex vivo, aiming to support their suitability for intravesical administration. The cytotoxicity assays performed on MRC-5 fibroblasts revealed that none of the tested treatments achieved a selectivity index (SI) greater than 1.0, which is generally considered indicative of desirable selectivity (Table 4) [61]. Nevertheless, it is important to highlight that the nanospheres demonstrated an improved selectivity profile compared to free lapachol. This enhancement was not observed with the nanoemulsion, emphasizing the potential of the nanosphere formulation as a safer and more effective nanocarrier for bladder cancer treatment.

Fibroblasts are widely recognized as a suitable model for evaluating the selectivity of antitumor candidates due to their ubiquitous presence in most organs [62]. However, it is important to note that the MRC-5 cell line, although classified as non-tumorigenic, is an immortalized fibroblast line. Previous studies have shown that MRC-5 cells exhibit greater sensitivity to cytotoxic agents compared to primary normal cells [63]. This increased sensitivity may stem from alterations in membrane composition or function associated with the immortalization process [63]. Therefore, despite the low selectivity index observed with MRC-5 cells, the observed improvement in selectivity with lapachol-loaded nanospheres relative to the free drug is promising. Further investigations using primary urothelial or bladder endothelial cells are recommended to more accurately assess the safety and selectivity of the nanosphere formulation for intravesical therapy.

**Figure 8:**
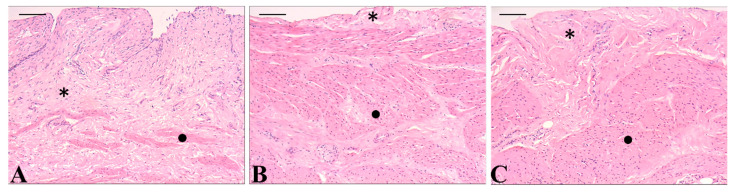
Photomicrographs of histological sections of pig bladder (*Sus scrofa domesticus*) treated with ex vivo formulations. (**A**): control; (**B**): nanospheres, and (**C**): nanoemulsion with lapachol. Hematoxylin–eosin. Bar = 50 µm. * Lamina propria and • muscular layer.

The ex vivo evaluation of lapachol-loaded chitosan nanoparticles further supports the continued investigation of these formulations. Qualitative histopathological analysis revealed no observable morphological alterations in bladder tissues treated with either formulation when compared to the control group (Figure 8). The lamina propria, consisting of loose connective tissue itself, and the muscular layer retained their characteristics after treatment with nanoemulsion or nanospheres. These findings suggest that the formulations did not induce tissue damage, indicating a favorable safety profile for intravesical administration [37,64]. Consequently, both nanostructured systems may be considered biocompatible and suitable for further preclinical development.

## 4. Conclusions

The chitosan-based nanoemulsion and nanospheres encapsulating lapachol exhibited appropriate particle sizes (around 200 nm) and high encapsulation efficiency (>90%). Furthermore, the chemical stability of lapachol was maintained throughout the preparation process of both nanoparticle types. These nanostructured systems enhanced the antiproliferative activity of lapachol against bladder tumor cells, reducing the CC_50_ by up to six-fold. Compared to the nanoemulsion, the nanospheres demonstrated superior cellular uptake and, consequently, greater cytotoxicity. Moreover, the nanospheres increased the selectivity index of lapachol, and histopathological analysis in the ex vivo model revealed no alterations, indicating the safety of the formulations when administered via the intravesical route. Therefore, chitosan-based nanoparticles containing lapachol show promise for the treatment of bladder cancer, and further preclinical in vivo and clinical studies are warranted.

## 5. Patents

Brandão: G.C., Amparo, T.R., Seibert, J.B., Assunção, K.F., Nanopartículas de quitosana contendo lapachol para tratamento de câncer de bexiga: método de obtenção, composição farmacêutica, caracterização e usos. Universidade Federal de Ouro Preto. Patent BR 10 2024 021600 8, 17 October 2024.

## Figures and Tables

**Table 1:** Validation parameters for lapachol quantification.

Linearity	
r^2^	0.9992
Equation	y = 0.0024x + 0.1075
Significance	*p* = < 0.0001 (a ≠ 0)
Linearity	*p* = 1.0000 (linear)
Precision (repeatability)	
Concentrations (µg mL^−1^)	RSD
100	2.99
200	1.81
400	1.68
500	2.04
800	1.56
1000	1.13
Accuracy	
Concentrations (µg mL^−1^)	RSD
100	2.99
500	2.04
1000	1.13

**Table 2:** Adjusted kinetic coefficient (R^2^) obtained by zero order, first order, Higuchi model, and Korsmeyer–Peppas model.

Sample	Kinetic Models (R^2^)			
	Zero Order	First Order	Higuchi	Korsmeyer–Peppas
Nanospheres	0.5709	0.4760	0.7672	0.8820
Nanoemulsion	0.4454	0.2715	0.6381	0.7200

**Table 3:** Cytotoxicity of the nanoemulsion, nanospheres, and lapachol against bladder cancer cell lines.

	Cell Lines (CC_50_ µg/mL)		
	T24	J82	RT4
Free lapachol	42.9 ± 6.1 ^a^	35.9 ± 2.0 ^a^	32.7 ± 3.8 ^a^
Nanospheres	6.5 ± 1.4 ^b^	10.1 ± 1.3 ^b^	14.7 ± 1.8 ^b^
Nanoemulsion	9.4 ± 1.4 ^b^	29.0 ± 3.7 ^b^	38.4 ± 9.1 ^a^

CC_50_: Cytotoxic concentration for 50% of cells expressed as equivalent to lapachol concentration. Same lowercase letters indicate no significant statistical difference in the same column (*p* < 0.05) by One-way ANOVA test followed by Tukey’s post-test.

**Table 4:** Cytotoxicity for fibroblasts MRC-5 and selectivity of the nanoemulsion, nanospheres, and lapachol against bladder cancer cell lines.

	MRC-5 CC_50_ (µg/mL)	Selectivity Index		
		T24	J82	RT4
Free lapachol	15.7 ± 0.7 ^a^	0.37 ± 0.05 ^a^	0.44 ± 0.02 ^a^	0.48 ± 0.06 ^a^
Nanospheres	6.2 ± 0.9 ^b^	0.99 ± 0.20 ^b^	0.62 ± 0.08 ^b^	0.43 ± 0.05 ^b^
Nanoemulsion	5.5 ± 0.2 ^b^	0.60 ± 0.09 ^a^	0.19 ± 0.03 ^b^	0.15 ± 0.03 ^a^

CC_50_: Cytotoxic concentration for 50% of cells expressed as equivalent to lapachol concentration. Same lowercase letters indicate no significant statistical difference in the same column (*p* < 0.05) by One-way ANOVA test followed by Tukey’s post-test.

## Data Availability

Data are contained within the article.
